# Antidiarrheal, antimicrobial and antioxidant potentials of methanol extract of *Colocasia gigantea* Hook. f. leaves: evidenced from *in vivo* and *in vitro* studies along with computer-aided approaches

**DOI:** 10.1186/s12906-021-03290-6

**Published:** 2021-04-12

**Authors:** Safaet Alam, Mohammad A. Rashid, Md. Moklesur Rahman Sarker, Nazim Uddin Emon, Mohammad Arman, Isa Naina Mohamed, Mohammad Rashedul Haque

**Affiliations:** 1 Department of Pharmaceutical Chemistry, Faculty of Pharmacy, University of Dhaka, Dhaka, 1000 Bangladesh; 2 Department of Pharmacy, State University of Bangladesh, 77 Satmasjid road, Dhanmondi, Dhaka, 1207 Bangladesh; 3 Department of Public Health, School of Science and Technology, Bangladesh Open University, Gazipur, Dhaka, 1705 Bangladesh; 4 Department of Pharmacy, International Islamic University Chittagong, Chittagong, 4318 Bangladesh; 5 Department of Pharmacology, Faculty of Medicine, Universiti Kebangsaan Malaysia (The National University of Malaysia), Cheras, Malaysia

**Keywords:** *Colocasia gigantea*, Human kappa-opioid receptor, Antioxidant, Molecular docking, Drug likeliness

## Abstract

**Background:**

*Colocasia gigantea*, locally named as kochu is well-known due to its various healing power. This research is to investigate the antidiarrheal, antimicrobial and antioxidant possibilities of the methanol soluble extract of *Colocasia gigantea*.

**Methods:**

The antidiarrheal investigation was performed by using in vivo castor oil-induced diarrheal method whereas in vitro antimicrobial and antioxidant investigation have been implemented by disc diffusion and DPPH scavenging method respectively. Moreover, in silico studies were followed by molecular docking analysis of several secondary metabolites that were appraised with Schrödinger-Maestro v11.1 and Biovia Discovery Studio.

**Results:**

The induction of plant extract (200 and 400 mg/kg, b.w, p.o) has minimized the castor oil mediated diarrhea by 16.96% (*p* < 0.01) and 38.89% (*p* < 0.001) respectively compared to control group. The methanol extract of *C. gigantea* showed mild sensitivity against almost all the tested strains but it shows high consistency of phenolic content and yielded 67.68 μg/mL of IC_50_ value in the DPPH test. In the PASS prediction, selected isolated compounds have demonstrated significant antidiarrheal and antimicrobial activity following the Lipinski drug rules which have ascertained efficacy with the compounds in molecular docking study.

**Conclusion:**

The results of this scientific research reflects that the methanol soluble extract of *C. gigantea* is safe and may provide possibilities of alleviation of diarrhea along with being a potential wellspring of antioxidant and antimicrobial agents which can be considered as an alternate source for exploration of new medicinal products in near future.

## Background

Diarrhea, a common disease in tropical countries and can be interpreted as an incidence of daily stool exceeding 200 g comprised of 60 to 95% of water. Infants and children suffer from diarrhea most, and mortality from diarrhea is high compared with other diseases [1]. Unhygienic living style plays a key role in making people lying to diarrhea. Various enteropathogens like *Escherichia coli, Shigella flexneri, Salmonella typhi*, *Staphylococcus aureus,* and *Candida albicans* are major causative agents that can provoke diarrhea [2]. As pathogenic bacterias result in a major cause of morbidity and mortality in humans, pharmaceutical companies are determined to produce masses of new antibacterials which are becoming significant against infections and drawing global concern [3]. A couple of justifications make clinical microbiologists interested in antimicrobials from plant extracts including the possibility of phytochemicals to be the arsenal of antimicrobial agents prescribed by the physicians and making people aware of the risks with the misuse of traditional antibiotics [4]. Oxidative stress, induced by oxygen radicals, is believed to be a primary factor in various deteriorating diseases, such as cancer [5] atherosclerosis [6], gastric ulcer [7], and other conditions. There is a rising interest in natural antioxidants which are derived from plants as bioactive components. The importance of the antioxidant constituents of plant materials in the maintenance of health and protection from coronary heart disease and cancer is also raising attention among the scientists, food manufacturers, and consumers as the trend of the future is moving toward functional food with specific health effects [8]. Many antioxidant compounds, naturally occurring from plant sources, have been identified as free radicals or active oxygen scavengers [9]. Recently, interest has increased considerably in finding naturally occurring antioxidants to use in foods or medicinal materials to replace synthetic antioxidants, which are being restricted due to their side effects such as carcinogenicity [10]. Healing with medicinal plants is as old as mankind itself [11]. Natural products derived from plants for the treatment of diseases have proved that nature stands as a golden mark to show the interrelationship between man and the environment [12]. 80% of drug substances are either a direct derivative of the natural component or a refined version of the natural part of the plant extracts [13]. The genus *Colocasia* is represented by 13 species worldwide [14] among which eight species were found in Asia and the Malay Archipelago initially [15]. In Bangladesh, so far nine species of the genus Colocasia are: *C. gigantea* (Blume) Hook. f., *C. fallax* Schott, *C. affinis* Schott, *C. esculenta* (L.) Schott, *C. oresbia* A. Hay, *C. heterochroma* H. Li et Z.X. & Wei*, C. virosa* Kunth, *C. lihengiae* C.L. Long et K.M. Liu, and *C. mannii* Hook. f [16].. *Colocasia* is a flowering plant genus under Araceae family native to southeastern Asia and the Indian subcontinent which are widely cultivated and naturalized in other tropical and subtropical regions [17]. In Fiji, the locals make use of either boiled or baked breadfruit or tubers of taro as slices along with roasted pig. Along with culinary items of taro *C. gigantea* has been used as medicine to treat constipation and tuberculosis in Hawaii [18]. Genus of *Colocasia* leaves has demonstrated the potentiality of demonstrating antidiabetic, antihypertensive, immunoprotective, neuroprotective, and anticarcinogenic activities [19]**.** *Colocasia gigantea* (Family: Araceae) is a perennial herb of 1.5–3 m tall available in South-East Asia and leaf stalk is consumed as a vegetable [20]. *C. gigantea* is abundantly found in Bangladesh and locally known as Kochu. This plant is also known as giant elephant ear or indian taro. Phytochemical extraction and structure elucidation of *Colocasia* leaves yield chemical compounds such as isoorientin, orientin, isoschaftoside, Lut-6-C-Hex-8-C-Pent, vicenin, alpha-amyrin, beta-amyrin, monoglyceryl stearic acid, penduletin, anthraquinones, apigenin, catechins, cinnamic acid derivatives, vitexin, and isovitexin [19, 21, 22]. A clinical trial is necessary in order to explain the final effectiveness of a medication or drug source, but the trial requires moving through the preclinical stage before entering into the clinical trial phase [23]. The compatibility between animal trials and clinical trials of a drug may vary in some cases but the findings of animal trials can be correlated with clinical trial owing to matching genomic factors [24]. Besides, only in vitro testing is not enough to make a strong conclusion regarding antidiarrheal property [25]. Considering all these points, animal trial has been implemented where the mice was used as animal models to evaluate antidiarrheal action while disc diffusion method was applied to evaluate antibacterial action and total phenol content along with free radical scavenging activity was performed to assess antioxidant property. Several ethnomedical studies were published, including therapeutic benefits of cancer and tumor activities of this plant [26]. The use of *in vitro*, *in vivo* and *in silico* models gives insights into the efficacy of the plants for the treatment of several maladies and nature of the plants for further studies [27]. However, until now, no antidiarrheal, antibiotic and antioxidants activity studies of MECG have been carried out using modern pharmacological techniques. Therefore, considering all auspicious factors, this study is conducted to evaluate the antidiarrheal, antimicrobial, and antioxidant activity of methanol extract of *C. gigantea* by biological and computational approaches*.* It must also be tested by means of structured laboratory procedures to ensure safety and care.

## Methods

### Collection and extraction of plant

The leaves of *C. gigantea* were collected from Bandarban in May 2019. The plant was identified by the experts of Bangladesh National Herbarium, Mirpur, Dhaka and a voucher specimen (DACB; Accession no: 57066) has been deposited for this collection. After proper washing, the leaves of *C. gigantea* were sun-dried for several days. The dried plant was then grounded to a coarse powder by using a high capacity grinding machine. Eight hundred grams of the powdered material was taken in a clean, and round bottom flask (5 l) and soaked in 2.4 l of methanol. The container with its content was kept for a period of 10 days accompanying daily shaking and stirring. The whole mixture was then filtered through a fresh cotton plug and finally with a Whatman No.1 filter paper. The volume of the filtrate was then reduced by using a Buchi Rotavapor at low temperature and pressure. The weight of the crude extract was found 60.82 g.

### Drugs and chemicals

All drugs and chemicals used in this research were of analytical grade. Methanol, Tween-80, was purchased from Merck (Darmstadt, Germany). 1,1-diphenyl-2-picrylhydrazyl radical (DPPH), Folin-Ciocalteau reagent (FCR) were obtained from Sigma Chemicals Co. (St. Louis, MO, USA). Loperamide (Square Pharmaceuticals Ltd., Dhaka, Bangladesh); Amoxicillin (Beximco Banglades Ltd., Dhaka, Bangladesh); were procured from the mentioned sources.

### Experimental animals

Swiss-albino mice of either sex, aged 4–5 weeks, acquired from the Animal Resource Branch of the International Centre for Diarrhoeal Diseases and Research, Bangladesh (ICDDR,B) and were used for the experiment. They were housed in standard polypropylene cages and kept under controlled room temperature (24 ± 2 °C; relative humidity 60–70%) in a 12 h light-dark cycle and fed ICDDR,B formulated rodent food and water (ad libitum). As these animals are very sensitive to environmental changes, they were kept in the environment where the experiment will take place before the test for at least 3–4 days. The experiments were conducted in accordance with the guideline for the care and use of laboratory animals. The protocols for conducting the experiments on the animals were permitted by the institutional ethical committee [28]. The Animal Ethics Committee of the State University of Bangladesh, Dhaka, Bangladesh, has issued the ethical approval (Approval Number: 2019-08-26/SUB/A-ERC/0012). All conducted experiments were performed by following the endorsed Animal Use Protocol by the Ethics Committee and in compliance with the Guidelines issued by the US National Institutes of Health for the Care and Use of Laboratory Animals. The guidelines and recommendations of the Federation of European Laboratory Animal Science Associations (FELASA) were implemented to ensure the reduction of pain and stress of the laboratory models. During the designing of the reseach and experiments “3R” (Replace, Reduce and Refine) was strictly maintained and to avoid severe pain and sufferings experienced and trained researchers and laboratory assistants handled the experiment. At the end of the experiment, an anesthesia overdose (Ketamine HCl (100 mg/kg) and Xylazine (7.5 mg/kg) through the intraperitoneal route was given to the mice models followed by euthanasia [29, 30].

### In vivo oral acute toxicity test

The oral acute toxicity test was conducted under normal conditions in laboratories and followed the “Organization for Environmental Control Development” guidelines (OECD: Guidelines 420) fixed-dose Method [31, 32] and after the administration of high oral doses (2000 mg/kg) to the mice, several parameters were recorded throughout the 72 h. As a result, there was no lethality, no behavioral change (sedation, excitability), or no allergic reaction was appeared after the oral administration methanol soluble leaves extract of *C. gigantea*. Regarding the safe dose adjustment from the view of oral acute toxicity, the doses 200 and 400 (mg/kg, b.w; p.o) has been choosen for the antidiarrheal activity study.

### In vivo castor oil induced diarrhea

The anti-diarrheal activity of the methanolic extract of leaves of *C. gigantea* was evaluated using the method of castor oil-induced diarrhea in mice followed by the method as described by Rudra et al [33]. According to this method, each mouse was fed with 1 mL of the highly pure analytical grade of castor oil which would induce diarrhea. The numbers of fecal stools were recorded for each mouse. The observations of the experimental groups were compared against that of the control to evaluate the anti-diarrheal activity of the samples. The animals were divided into control, positive control, and test groups containing five mice in each group. Control group received vehicle (1% Tween 80 in water) at dose 10 mL/kg orally. The positive control group received loperamide at the dose of 2 mg/kg orally. The test group received a methanolic extract of leaves of *C. gigantea* the doses of 200 and 400 (mg/kg, b.w; p.o). Each animal was placed in an individual cage; the floor lining was changed at every hour. Diarrhea was induced by oral administration of castor oil to each mouse after the above treatment. During an observation period of 4 h; the number of diarrhoeic feces excreted by the animals was recorded.

### In vitro antibacterial assay

The antimicrobial assessment has been performed by following the disc diffusion method [34]. In this classical method, on nutrient agar medium uniformly seeded with the test microorganisms dried and sterilized filter paper discs (6 mm diameter) containing the test samples of known amounts are placed. Antibiotics diffuse from a confined source through the nutrient agar gel and create a concentration gradient. Standard antibiotic (Amoxicillin) discs and blank discs are used as a positive and negative control. To allow maximum diffusion of the test materials to surround media these plates are kept at low temperature (4 °C) for 16 to 24 h. For optimum growth of the organisms, the plates are then inverted and incubated at 37 °C for 24 h. The test materials having antimicrobial properties inhibit microbial growth in the media surrounding the discs and thereby yield a clear, distinct area defined as a zone of inhibition. The diameter of the zone of inhibition expressed in millimeters is then measured to determine the antimicrobial activity of the test agent [35]. Antimicrobial activity was evaluated on the clinically isolated strains of the following pathogens *Bacillus cereus, Bacillus megaterium, Bacillus subtilis, Staphylococcus aureus, Sarcina lutea* as gram-positive bacteria *and Escherichia coli, Pseudomonas aeruginosa, Salmonella paratyphi, Salmonella typhi, Shigella dysenteriae, Vibrio mimicus,* and *Vibrio parahemolyticus* as a gram-negative bacteria. Wells of 6 mm diameter were punched into the agar medium with sterile cork borer under aseptic conditions and filled with 50 μL of 250 mg/mL of plant extract, solvent blank, and standard antibiotic. Serial dilutions were prepared from 250 mg/mL of the plant extract using DMSO to make 250, 125, 62.5, 31.25, and 15.625 mg/mL. The wells were inoculated with 0.1 mL aliquot of test organisms (10^6^ CFU/mL) having serial dilutions of the extract (50 μL, each). Standard Amoxicillin (30 μg/disc) discs were used as a positive control to ensure the activity of standard antibiotics against the test organisms as well as for comparison of the response produced by the known antimicrobial agent with that produced by the test sample.

### In vitro total phenolic contents analysis

The total phenolic content of *C. gigantea* extractives was measured employing the method as described [36] involving Folin-Ciocalteu reagent as an oxidizing agent and gallic acid as standard. To 0.5 mL of extract solution (2 mg/mL), 2.5 mL of Folin-Ciocalteu reagent (diluted 10 times with water) and 2.0 mL of Na_2_CO_3_ (7.5% w/v) solution was added. The mixture was incubated for 20 min at room temperature. After 20 min the absorbance was measured at 760 nm by UV-spectrophotometer and using the standard curve prepared from gallic acid solution with different concentrations, the total phenols content of the sample was measured. The phenolic contents of the sample were expressed as mg of GAE (gallic acid equivalent)/gm of the extract.

### In vitro assay of free radical scavenging activity

DPPH was used to evaluate the free radical scavenging activity (antioxidant potential) of various compounds and medicinal plants [37]. 2.0 mL of a methanol solution of the sample (extractives/control) at different concentration (500 μg/mL to 0.977 μg/mL) were mixed with 3.0 mL of a DPPH methanol solution (20 μg/mL). After a 30 min reaction period at room temperature in a dark place, the absorbance was measured at 517 nm against methanol as blank by UV spectrophotometer. Tert-butyl-1-hydroxytoluene (BHT) has been used as positive control and observed with DPPH. Inhibition of free radical DPPH in percent (%) was calculated as follows:

$$ \%\mathrm{inhibition}=\frac{\left(1-\mathrm{Absorbance}\ \mathrm{of}\ \mathrm{sample}\right)}{\mathrm{Absorbance}\ \mathrm{of}\ \mathrm{blank}}\ \mathrm{X}\ 100 $$

Where A_blank_ is the absorbance of the control reaction (containing all reagents except the test material).

### Molecular docking analysis: selection of compounds for the computational study

**Fig. 1:**
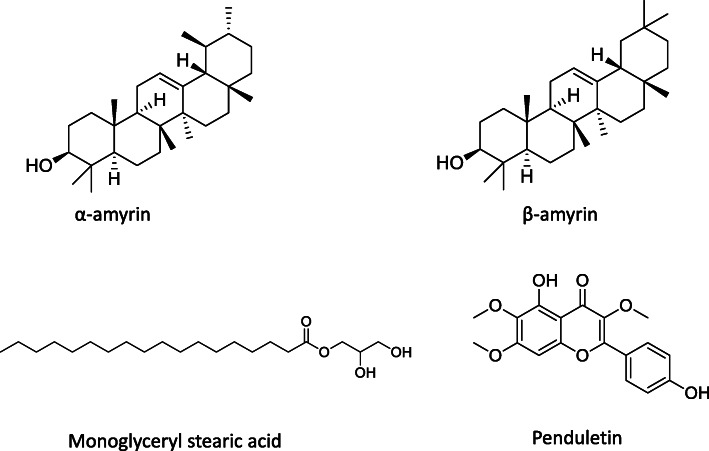
Chemical structures of Alpha-Amyrin, Beta-Amyrin, Monoglyceryl stearic acid and Penduletin used for the computational study

Alpha-amyrin (PubChem CID: 73170), beta-amyrin (PubChem CID: 73145), monoglyceryl stearic acid (PubChem CID: 24699) and penduletin (PubChem CID: 5320462) were selected based on the availability as major compounds through chemical investigation [19, 21, 22]. The chemical structures of isolated compounds have been retrieved from PubChem (https://pubchem.ncbi.nlm.nih.gov/) and presented in Fig. 1.

### Molecular docking analysis: ligand preparation

The chemical structures of the four compounds (alpha-amyrin, beta-amyrin, monoglyceryl stearic acid, and penduletin) of *C.gigantea* were downloaded from the PubChem compound database (https://pubchem.ncbi.nlm.nih.gov/). By means of the Lig Preptool which was incorporated in Schrödingersuite-Maestro v 11.1, the ligand was created where the following factors were used as follows: neutralized at pH 7.0 ± 2.0 using Epik 2.2 and the OPLS_2003 force field were used for minimization.

### Molecular docking analysis: enzyme/receptor preparation

3D structures of macromolecules have been obtained from the Protein Data Bank RCSBPDB [38] including kappa-opioid receptor (PDB: 6VI4) [39] and human delta-opioid receptor (PDB: 4RWD) [40] for antidiarrheal docking study, Beta-ketoaryl-ACP synthase 3 receptor (PDB: 1HNJ) [41] for antimicrobial docking study, Glutathione reductase (PDB: 3GRS) [42] and Urase oxidase (1R4U) [43] for antioxidant docking study. The enzyme/receptor was prepared for a docking experiment using Protein Preparation Wizard, which was embedded in Schrödinger suite-Maestro v11.1.

### Molecular docking analysis: glide standard precision docking

A molecular docking study was performed to reveal the possible mechanism of action of the selected compounds behind the biological activities of the *C. gigantea* against the respective enzymes/receptor for an antidiarrheal, and antibacterial activity. Docking experiments were performed using Glide standard precision docking, which was embedded in Schrödingersuite-Maestro v 11.1, as we described previously [44]. Finally, BIOVIA Discovery Studio Visualizer 2020 [45] has been accelerated to assesses docking positions for the best linking strategies.

### In Silico study: determination of pharmacokinetic parameters by SwissADME

The pharmacokinetic parameters or drug-likeness properties of the selected compounds were determined by SwissADME online total molecular weight of the compounds, Lipophilicity (LogP), the number of hydrogen-bond acceptors, and the number of hydrogen-bond donors based on the Lipinski’s rule.

### In Silico study: toxicological properties prediction by Admet SAR

The toxicological properties of the designated compounds have been determined by the admetSAR online tool (http://lmmd.ecust.edu.cn/admetsar1/predict/) since toxicity is a prime apprehension throughout the development of new drugs. The present study projected Ames toxicity, carcinogenic properties, and acute rat toxicity.

### PASS prediction study

The four major phytoconstituent Alpha-Amyrin, Beta-Amyrin, Monoglyceryl stearic acid Penduletin were investigated for evaluating the antidiarrheal, antibacterial, and antioxidant activities by using PASS online program.

### Statistical analysis

The data was presented as a standard error mean (SEM). Statistical analyses using single-way ANOVA were conducted and followed by Dunnett’s multiple comparison tests. The observed values were compared to the control group and were considered statistically significant at *p* < 0.05, *p* < 0.01, *p* < 0.001.

## Results

### Castor oil-induced diarrheal assay

**Fig. 2:**
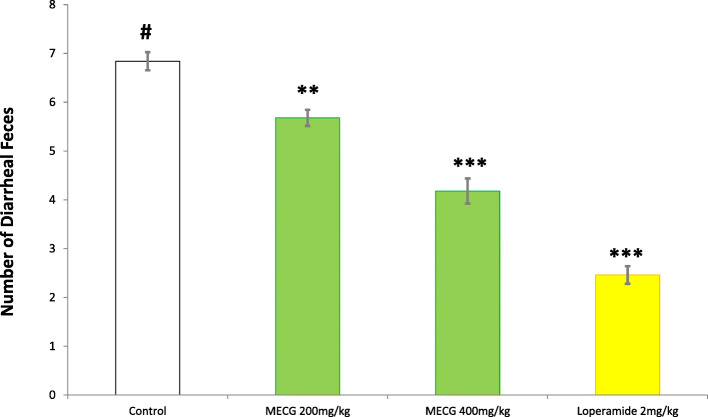
Effect of methanolic extract of leaves of *C. gigantea* on castor oil induced diarrhea in mice. Each value has been expressed as mean ± SEM (*n* = 5). *p < 0.05, **p < 0.01, ***p < 0.001 and compared with the control group (Dunnett’s test). TWN-80 = 1% tween – 80, MECG = methanol extract of *C. gigantea* leaves, LPM-2 = Loperamide 2 mg/kg

The methanol extract of *C. gigantea* leaves exhibited promising anti-diarrheal activity with a 16.96% (*p* < 0.01) and 38.89% (*p* < 0.001) reduction of diarrhea at the dose of 200 mg/kg and 400 mg/kg compared to the standard loperamide 64.04% which has extremely statistically significant anti-diarrheal activity. The result of this study shows that the methanol extract of *C. gigantea* possesses noteworthy anti-diarrheal activity which is dose-dependent and activity is more pronounced and statistically significant (*p* < 0.001) at 400 mg/kg body weight dose. The findings have been shown in Fig. 2.

### Analysis of antimicrobial activity

The methanol soluble extract of *C. gigantea* leaves exhibits mild inhibition against microbial growth having a zone of inhibition ranged from 11 mm to 18 mm. the maximum zone of inhibition produced by the methanol extract of *C. gigantea* leaves was found to be 18 mm against *Staphylococcus aureus* and *Salmonella typhi* followed by 17 mm against *Vibrio parahemolyticus* respectively. Besides, amoxicillin used as a standard drug exhibited prompt antimicrobial activity from 41 mm against *Staphylococcus aureus* and *Pseudomonas aerginosa*. The growth of inhibition of the microbe has been presented in Table 1.

**Table 1:** Antimicrobial activity of test samples of *C. gigantea* and Amoxicillin against gram positive and gram negative bacterial strains

Diameter of Zone of Inhibition (mm)		
Test Microorganisms	*C. gigantea*	Amoxicillin
Gram positive bacteria		
*Bacillus cereus*	13	37
*Bacillus megaterium*	12	35
*Bacillus subtilis*	11	34
*Staphylococcus aureus*	18	41
*Sarcina lutea*	14	37
Gram negative bacteria		
*Escherichia coli*	15	38
*Pseudomonas aeruginosa*	14	41
*Salmonella paratyphi*	16	30
*Salmonella typhi*	18	39
*Shigella dysenteriae*	16	37
*Vibrio mimicus*	13	30
*Vibrio parahemolyticus*	17	40

### Total phenolic content determination of *C. gigantea*

**Fig. 3:**
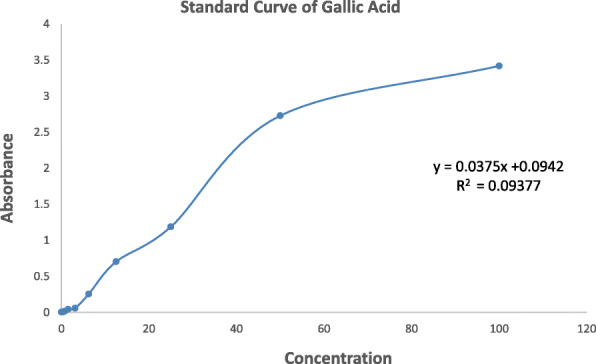
The curve of gallic acid for total phenolic contents determination of methanol extract of *C. gigantea*

The amount of total phenolic content for the methanol extract of *C. gigantea* leaves has been found 39.01 mg of GAE/gm of extractives. The phenolic contents of the sample were expressed as mg of GAE (gallic acid equivalent)/gm of the extract. The average phenolic content of standard (Gallic acid) and methanolic extract of *C. gigantea* has been shown in Table 2 and Fig. 3.

**Table 2:** Total phenolic content determination of methanol extract of *C. gigantea*

Sample	Absorbance 1	Absorbance 2	Average Absorbance	(mg/g of extract)
*C. gigantea*	1.546	1.568	1.557	39.01

### Free radical scavenging activity (DPPH)

**Fig. 4:**
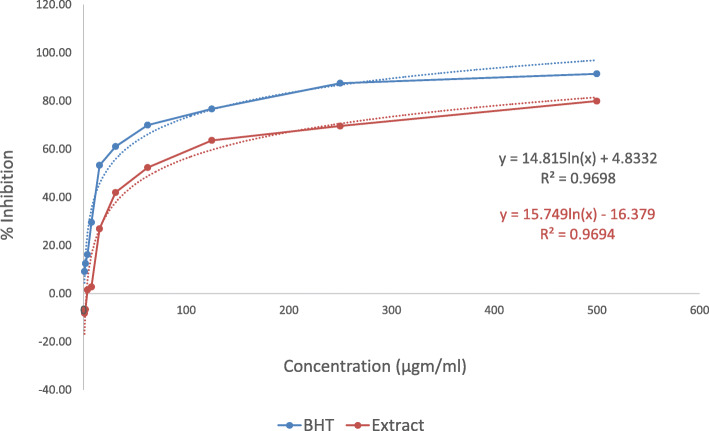
IC_50_ value of tert-butyl-1-hydroxytoluene (BHT) and methanol soluble extract of *C. gigantea* observed with DPPH

The IC_50_ values of methanol extract of *C. gigantea* leaves in the DPPH method have been compared to tert-butyl-1-hydroxytoluene (BHT) and the IC_50_ value has been found 67.68 μg/mL for the methanol soluble extract of *C. gigantea* and IC_50_ value for BHT was found 21.09 μg/mL. The summary of the IC_50_ values of the test sample and the tert-butyl-1-hydroxytoluene (BHT) have been presented in Fig. 4.

### Molecular docking study: antidiarrheal activity

**Fig. 5:**
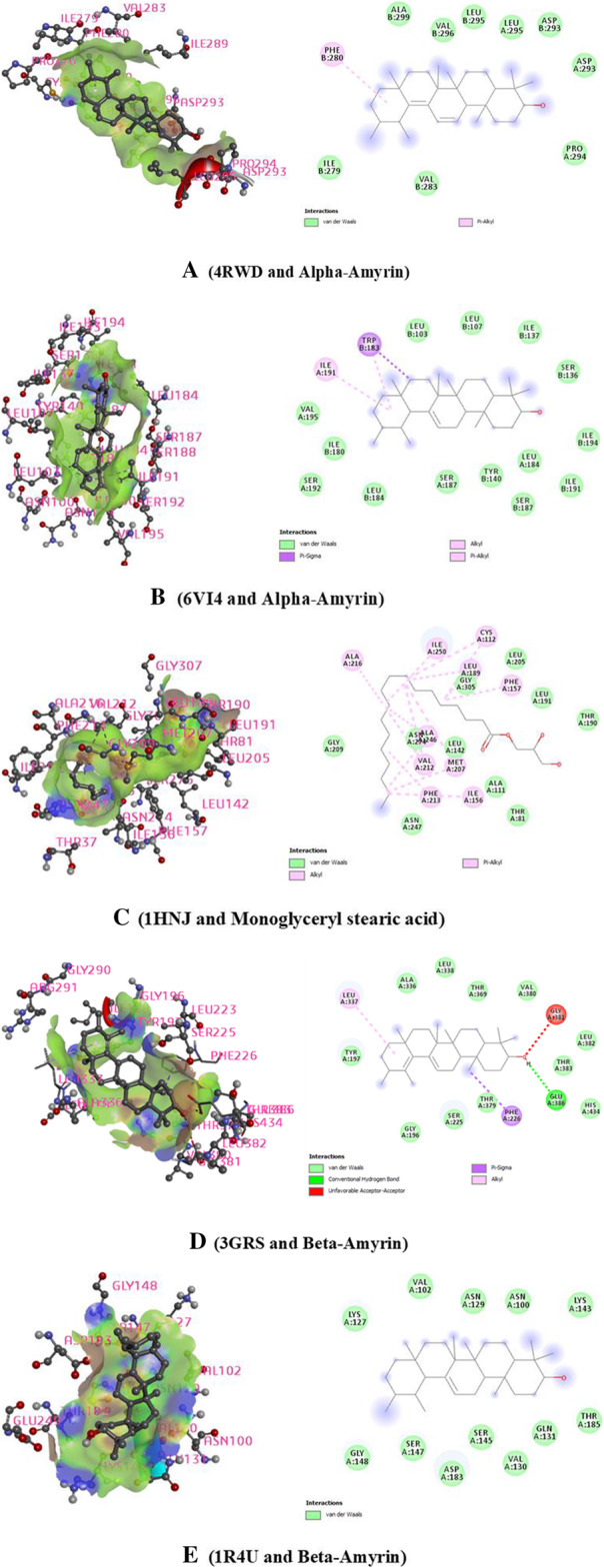
Presentation of the best binding interactions (2D and 3D) of **a** (4RWD and Alpha-Amyrin), **b** (6VI4 and Alpha-Amyrin), **c** (1HNJ and Monoglyceryl stearic acid), **d** (3GRS and Beta-Amyrin), **e** (1R4U and Beta-Amyrin)

In this case, beta-amyrin and monoglyceryl stearic acid have demonstrated the maximum and lowermost binding affinity against the human kappa-opioid receptor (PDB ID: 6VI4) varies with a docking score of − 4.3 to − 7.9 (kcal/mol). Alpha-amyrin yielded the highest binding affinity to the 6VI4 receptor through the interaction of a series of amino acid residues (val283, ile279,phe280, ala299, val296, leu295, asp293, asp293 and pro294). The ranking order of the docking score is presented as follows: Alpha-Amyrin > Loperamide > Beta-Amyrin > Penduletin > Monoglyceryl stearic acid. Human delta-opioid receptor (PDB ID: 4RWD) fluctuates with a docking score of − 8.4 to − 10.3 (kcal/mol) respectively. The ranking order of the docking score is presented as follows: Alpha-Amyrin > Loperamide > Monoglyceryl stearic acid > Beta-Amyrin > Penduletin. Loperamide (reference drug) showed a docking score − 6.6, and − 10.1 (kcal/mol) to the kappa and delta-opioid receptor respectively (PDB ID: 6VI4, and 4RWD) (Table 3 and Fig. 5).

**Table 3:** Docking scores or binding affinity of the selected compounds with the kappa-opioid receptor (PDB: 6VI4), human delta-opioid receptor (PDB: 4RWD), Beta-ketoaryl-ACP synthase 3 receptor (PDB: 1HNJ), Glutathione reductase (PDB: 3GRS), Urase oxidase (1R4U) for the antidiarrheal, antibacterial and antioxidant activity respectively

Docking Score						
		Antidiarrheal		Antibacterial	Antioxidant	
Compounds	PubChem ID	4RWD	6VI4	1HNJ	3GRS	1R4U
Standard (Loperamide/Amoxicillin/Ascorbic acid)	3955/ 2764/54670067	−6.6	−10.1	−4.5	−6.0	−5.4
Alpha-Amyrin	73170	**−7.9**	**−10.3**	−4.5	−10.1	−6.7
Beta-Amyrin	73145	−7.2	−9.5	–	**−10.4**	**−7.1**
Monoglyceryl stearic acid	24699	−4.3	−9.8	**−6.1**	−5.5	− 4.6
Penduletin	5320462	−5.4	−8.4	−5.7	−7.4	−6.1

### Molecular docking study: antibacterial activity

In the antimicrobial investigation, the monoglyceryl stearic acid and alpha-amyrin obtained the highest and lowest binding affinity against the Beta-ketoaryl-ACP synthase 3 receptor with a docking score of − 4.5 kcal/mol and − 6.1 kcal/mol. Monoglyceryl stearic acid bonded to the Beta-ketoaryl-ACP synthase 3 receptor throughout the bonds where the bonds has been formed due to the series of amino acid residues (van der waals: asn247, gly209, asn274, gly305, leu205, leu191, thr190, ala111, thr81. Alkyl: ile156, phe213, met207, val212. Pi-Alkyl: ala216, ile250, leu189, cys112, phe157). The ranking order of the docking score is presented as follows: Monoglyceryl stearic acid > Penduletin > Beta-Amyrin > Amoxicillin > Alpha-Amyrin. The results of the docking study has been showed in Table 3 and Fig. 5.

### Molecular docking study: antioxidant activity

In this study, Glutathione reductase (PDB: 3GRS) and Urase oxidase (1R4U) was docked with the selected compounds thus to assess the binding interactions. Beta-Amyrin showed a prominent binding affinity with both Glutathione reductase (PDB: 3GRS) and Urase oxidase (1R4U) enzymes. Beta-Amyrin interact to the 3GRS receptor via a series of amino acid residues: van der waals (leu184, ser192, ile180, val195, ieu103, ieu107, ile137, ser136, ile194, ile191, leu184, ser187, tyr140, ser187), pi-Sigma (trp183). alkyl (ile191). The ranking of the docking score is as follows: Beta-Amyrin > Alpha-Amyrin > Penduletin > Monoglyceryl stearic acid > Ascorbic acid. Besides, the binding interactions of compounds and Urase oxidase (1R4U) is as follows: Beta-Amyrin > Alpha-Amyrin > Penduletin > Ascorbic acid > Monoglyceryl stearic acid. Beta-Amyrin binds to the Urase oxidase via a series of bonds: van der waals (val283, ile279, ala299, val296, ieu295, leu295, asp293, pro294), pi-Alkyl (phe280). The summaries of the docking study are shown in Table 3 and Fig. 5.

### Pharmacokinetic (ADME) and toxicological properties

Prediction The pharmacokinetic features of the substances chosen by Lipinski were determined using SwissADME, the online tool. Lipinski has here declared that if a drug/compound follows the following criteria such as molecular weight < 500 amu, Hydrogen bond donor sites < 5, Hydrogen bond acceptor sites < 10, and Lipophilicity value LogP ≤5, then the compound would be orally bioavailable. The study showed that all the compounds complied with the rules of Lipinski, suggesting the strong oral bioavailability of these compounds (Table 4). Besides, the admetSAR online server predicted the toxicological properties of the four selected compounds. The analysis revealed that the selected compounds are non-Ames toxic, non-carcinogenic, and had low rat toxicity values.

**Table 4:** Physicochemical and toxicological properties of the compounds for good oral bioavailability

Compounds	Molecular Weight (M.W) (g/mol)	H-bond Donor	H-bond Acceptors	Lipophilicity - log P (o/w)	GI Absorption	AMES Toxicity	Carcinogens	Rat Acute Toxicity
Loperamide	477.04	1	3	4.13	High	No	No	3.6560
Amoxicillin	365.40	4	6	1.22	Low	No	No	1.7036
Alpha-Amyrin	426.72	1	1	4.77	Low	No	No	2.0842
Beta-Amyrin	426.72	1	1	4.75	Low	No	No	2.0842
Monoglyceryl stearic acid	358.56	2	4	4.59	High	No	No	0.8172
Penduletin	344.32	2	7	2.84	High	No	No	3.1579

### PASS prediction study

Four major selected compounds of *C. gigantea* were studied by the PASS online tool for antidiarrheal, antibacterial and antioxidant activities. The potentcy displayed higher Pa value than Pi (Table 5).

**Table 5:** PASSS prediction of standard drug and selected bioactive compounds of *C.gigantea*

BiologicalActivity	Chemical Constituents									
	Standard (Loperamide,Amoxicillin and Ascorbic acid)		Alpha-Amyrin		Beta-Amyrin		Monoglyceryl stearic acid		Penduletin	
	Pa	Pi	Pa	Pi	Pa	Pi	Pa	Pi	Pa	Pi
Antidiarrheal	0.574	0.003	0.085	0.011	0.353	0.023	0.754	0.003	0.588	0.006
Antibacterial	0.761	0.003	0.200	0.117	0.206	0.112	0.291	0.06	0.359	0.041
Antioxidant	0.928	0.003	0.411	0.011	0.405	0.01	0.276	0.028	0.714	0.004

## Discussion

Several steps including detection and characterization of bioactive substances are required to address the therapeutic activity of medicinal plants [46]. Even without a clear concept of proper dosing profile, indigenous people exploit medicinal plants in various forms including pastes, juices, or boiled leaf extracts which makes researchers curious about exposing the pharmacologic potentiality by the efficient solvent extraction process. A number of bioactive compounds like flavonoids, alkaloids, tannins, alkaloids, terpenoids, etc. are quickly miscible in methanol due to high polarity index of methanol (5.1). This justifies the wide use of methanol in the extraction process of medicinal plants which is also used in the investigation of *C. gigantea* [47]. Several studies on the treatment of a number of pathological disorders including diarrhea, microbial, and oxidative degradations have been conducted using noble molecules due to their lower side effects [48]. This proximate study includes the pharmacological assessment of the methanol extract of *C. gigantea* leaves by maneuvering isolated bioactive phytochemicals to aim antidiarrheal, antimicrobial, and antioxidant effects followed by computational analysis (in silico molecular docking, ADME and toxicity predictions) of it’s isolated bioactive compounds.

In this study, the methanol extract of *C. gigantea* leaves was found to show significant (*p* < 0.01 and 0.001) dose-dependent inhibition of the frequency of diarrheal feces. Lack of harmony between intestinal smooth muscle motility and/or absorption pattern of the GI tract can inaugurate diarrhea [49]. The usage of castor oil as a diarrhea medication has been well recognized [50]. Castor oil, a very nifty laxative which has been used as diarrhea inducer owing to promote hydrolyzation in the upper small intestine to ricinoleic acid and able to provoke fluid secretion, inhibit water and electrolyte absorption, reduce active Na^+^ and K^+^ absorption, and decrease Na^+^, K^+^, −ATPase in the small intestine and colon [51] which is accomplished by the irritant effect of ricinoleic acid liberated by pancreatic acid [52]. Prostaglandins which possess a basic role in the pathophysiology of diarrhea can be released by ricinoleic acid that regulates the gastrointestinal tract, stimulate motility secretion and eventually causes diarrhea [53, 54]. Recently revealed molecular mechanism suggests the activation of EP3 prostanoid receptor by ricinoleic acid which orients the pharmacologic effects of castor oil. Intestinal and uterine-muscle cells have been triggered by ricinoleic acid via EP3 prostanoid receptors which illuminate the cellular and molecular mechanism of castor oil inducing laxative outcome of castor oil. The antidiarrheal property of methanol extract of *C. gigantea* may be exhibited via several mechanisms including the reduction of prostaglandin secretions [55]. Flavonoids and alkaloids containing plant extracts modify cyclo-oxygenase 1 and 2 (COX-1, COX-2) and lipo-oxygenase (LOX) production which obstructs prostaglandin and autacoids production [56]. Flavonoids, a large group of polyphenolic compounds holding a wide variety of biological effects such as antioxidant, anti-inflammatory, antispasmodic, and antidiarrheal activities [57] can also display the antidiarrheal activity too by restricting intestinal motility and hydro-electrolytic secretions [58].

Antimicrobial property by an extract depends on the phytochemical composition, extracting solvent, effective solubility and miscibility of the active component in the test medium, the vulnerability of the test organisms, and the method used in evaluation [59]. Previous research suggests a bunch of phytochemical compounds like glycoside, saponin, tannin, flavonoids, terpenoid, and alkaloids as antimicrobial entities [60]. Antibacterial agents can disrupt the bacterial cell wall resulting in cytoplasm leakage and coagulation in gram positive bacteria. Like others, phytocompounds found in *C. gigantea* leaves methanol extract could be involved in a number of bacterial biosynthetic pathways like cell wall, DNA, lipid, and/or protein synthesis inhibitors [48]. This study was propagated to assess the antimicrobial effect of the methanol extract of *C. gigantea* leaves and from the investigation, it was noticed that *C. gigantea* is mildly susceptible to the test strains. Therefore, the methanol extract of *C. gigantea* leaves may be considered as a source of antimicrobial moieties for further researches.

Plant extracts that are rich in polyphenols can exhibit redox properties by absorbing and neutralizing free radicals utilizing scavenging properties of their hydroxyl group and demonstrate antioxidant activity [61, 62]. Among those, the flavonoid is considered as supremely competent scavengers of most oxidizing molecules, including quenching single and triplet oxygen or decomposing peroxides and various free radicals implicated in several disease conditions [63]. *C. gigantea* showed a concentration-dependent antiradical activity by inhibiting DPPH radical with an IC_50_ value of 67.68 μg/mL and the total phenolic compound was determined 39.01 mg of GAE/ gm of crude extract. The result promotes that, *C. gigantea* possesses dose-dependent hydrogen donating capabilities and acts as a prominent source of antioxidant. In the end of enumerating all upshots, our denouement suggested that alkaloids, phenol, flavonoids, terpenoids, tannins, etc.; maybe the major contributors to the antidiarrheal, antibacterial, and antioxidant activities of methanol soluble leaves extract of *C. gigantea*.

Molecular docking analyses were employed extensively in the estimation of ligand-target relationships and to gain a deeper understanding of the biological activity of natural products. It provides more insights into probable mechanisms of action and binding mode within the binding pockets of several proteins [64]. Four isolated compounds within *C.gigantea* have been selected for docking tests to provide greater insight into the biological activity (antidiarrheal and antibacterial). These compounds were then docked against seven targeted receptors, namely the kappa opioid receptor (PDB: 6VI4), human delta-opioid receptor (PDB: 4RWD), Beta-ketoaryl-ACP synthase 3 receptor (PDB: 1HNJ), Glutathione reductase (PDB: 3GRS) and Urase oxidase (1R4U). Docking score revealed that, among the four compounds, interacted with enzymes through several amino acid residues and formed the docking scores ranging from − 4.3 to − 10.4 kcal/mol. From these results, we can conclude that the studied phytoconstituents may in part be responsible for the antidiarrheal, antimicrobial and as well as antioxidant activities of *C.gigantea*.

The online prediction software ADME has been used to investigate the drug-like properties, pharmacokinetics, and physicochemical characteristics in all bioactive compounds. According to Lipinski’s law, all the bioactive compounds exhibited orally active drug-likeness properties. Compounds with lower molecular weight, lipophilicity, and hydrogen bonding are stated to be highly permeable [65], good absorption, and bioavailability [66].

To assess the possible pharmacological profile of the compounds we used the structures based prevision system for biological activity, namely Prediction for Activity Spectra for Substances (PASS). The consequences recommended numerous activities, among these, we have found possible activity values (Pa range 0.085–0.754) for all four compounds for antidiarrheal, antibacterial, and antioxidant actions, supporting our laboratory investigations of methanol extract of *C. gigantea*. In addition, the wider ability of the species has been predicted by many other experiments. In summary, the use and detail observed effects of *C.gigantea* may be caused by the combined behavior of many phytoconstituents, both those reported herein and other compounds that have not yet been described.

## Conclusion

The outcome interpretation of this scientific study demonstrates that, the methanol extract of *C. gigantea* leaves can be a propitious wellspring of natural antioxidants along with an auspicious nominee for antidiarrheal and mild antimicrobial treatment. Additionally, in molecular docking analysis, several bioactive promising biomolecules exhibited optimistic binding affinity to specific proteins, and the ADME/T study displayed their drug-like characters. Besides, the experimental findings have been steady with PASS predictions for bioactive constituents. However, additional studies are required to elucidate mechanism of actions of accountable bioactive phytocinstituents in order to support the present insights.

## Data Availability

All analyzed data during this research are included in the published manuscript. The generated datasets during this research is not publicly available though it can be providable from the corresponding author upon reasonable request.

## Abbreviations

DPPH: 1,1-diphenyl-2-picrylhydrazyl radical
FCR: Folin-Ciocalteau reagent
WHO: World Health Organization
ICDDR,B: International Centre for Diarrhoeal Diseases and Research, Bangladesh
OECD: Organization for Environmental Control Development
BW: Body weight
PO: Orally
PDB: Protein Data Bank
PASS: Prediction of activity of substance spectra
ANOVA: Analysis of variance
SEM: Standard error of the mean
BHT: Tert-butyl-1-hydroxytoluene
GAE: Gallic acid equivalent
ADME/T: Absorption, distribution, metabolism, excretion and toxicity
COX: Cyclo-oxygenase
LOX: Lipo-oxygenase
